# Lymphomatoid Granulomatosis Treated Successfully with Rituximab in a Renal Transplant Patient

**DOI:** 10.1155/2011/865957

**Published:** 2011-04-19

**Authors:** Cindy Castrale, Wael El Haggan, Françoise Chapon, Oumedaly Reman, Thierry Lobbedez, Jean Philippe Ryckelynck, Bruno Hurault de Ligny

**Affiliations:** ^1^Department of Nephrology and Renal Transplantation, Caen University Hospital, 14033 Caen, France; ^2^Laboratory of Pathology, Caen University Hospital, 14033 Caen, France; ^3^Department of Clinical Hematology, Caen University Hospital, 14033 Caen, France

## Abstract

Lymphomatoid granulomatosis (LYG) in renal transplant recipients is rare multisystemic angiocentric lymphoproliferative disorder with significant malignant potential. Here, we describe LYG in a 70-year-old renal allograft recipient who, 4 years after transplantation, on tacrolimus and mycophenolate mofetil and prednisone maintenance immunosuppression, complained of low-grade fever, persistent headache and gait disturbance. The MRI of the brain revealed diffuse periventricular cerebral and cerebellar contrast-enhanced lesions. The CT scan of the thorax showed multiple pulmonary nodular opacities in both lung fields. The patient was diagnosed LYG based on the cerebral biopsy showing perivascular infiltration of CD20-positive B-lymphocytes with granulomatous lesions and immunofluorescence staining with anti-EBV antibodies. With careful reduction of the immunossuppression combined with the use of rituximab, our patient showed a complete disappearance of LYG, and she is clinically well more than 4 years after the diagnosis, with good kidney function. No recurrence has been observed by radiological imaging until now. This is the first report of a durable (>4 years) complete remission of LYG after treatment with rituximab in renal transplantation.

## 1. Introduction

Posttransplant lymphoproliferative diseases (PTLD) are recognized as a main complication following solid organ transplantation. They generally occur in the 1st posttransplant year and may be triggered by Epstein-Barr virus (EBV) infection. Lymphomatoid granulomatosis (LYG) is a rarer disease, characterized by angiocentric and necrotising lymphoproliferation [1, 2] Its low prevalence and its lethal outcome did not allow to well establish an obvious treatment. We report here a case of a patient who had developed LYG with cerebral and pulmonary localization, treated successfully with rituximab. To our knowledge, this is the first case reported in renal transplantation.

## 2. Case Report

A 70-year-old woman, with renal failure secondary to chronic glomerulonephritis, had her 1st renal transplantation in 1993, after being on haemodialysis for 54 months. She was treated with an immunosuppressive regimen including lymphoglobuline, corticosteroids, azathioprine, and ciclosporine. Next, a transplantectomy was realized on day 15 as she developed *Candida glabrata* septicaemia. Her second transplantation was performed in 2001. The initial immunosuppressive treatment consisted of Thymoglobuline, corticosteroids, mycophenolate mofetil (MMF) and tacrolimus. Her serological tests for cytomegalovirus (CMV) and EBV were positive indicating previous infection, whereas that of toxoplasma was negative (negative IgG and IgM). Donor IgG of CMV and EBV were positive, in contrast, IgG and IgM of toxoplasma were negative. During the first posttransplant year, the patient presented CMV invasive infection with CMV-pneumonia; she was treated by IV Ganciclovir. After this episode, the patient was stable for almost 2 years. During the 4th year posttransplantation, she had presented multiple episodes of bronchopulmonary infection. Chest X-ray exams and CT scan did not show any abnormality. 54 months after transplantation, she had presented low-grade fever 38°C, posterior and temporal headache, progressive gait, and balance disorders, then a persistent cough. At this moment, her immunosuppressive treatment associated MMF(1,5 g/d), tacrolimus(1,5 mg/d), and prednisone(5 mg/d). The laboratory results revealed no elevated inflammatory markers, normal hepatic enzymes, and normal LDH; creatinine clearance according to MDRD formula was 42 mL/mn/1,73 m² and tacrolimus trough level was 5 *μ*g/l. 

**Figure 1:**
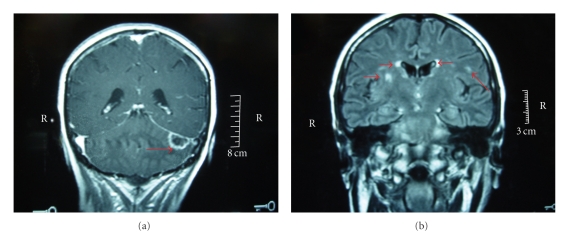
Cerebral MRI: (a) coronal view T1 with gadolinium injection: left cerebellar nodular lesion with central necrotic zone and peripheral contrast enhancement. (b) Coronal view T1 without gadolinium injection: periventricular localization of multiple cerebral hypersignal nodular lesions.

Analyses for EBV and CMV viruses by plasmatic PCR were negative. A lumbar puncture was performed; it revealed 8 cellular elements/mm^3^. The bacteriological, virological, mycological, and parasitological tests of the cerebrospinal fluid were all negative. The CT scan of the head without contrast injection was normal. The MRI performed 3 days later detected diffuse periventricular cerebral and cerebellar contrast-enhanced lesions (hypersignal Flair) (Figure 1). In view of these findings, the diagnosis of cerebral toxoplasmosis was considered. A reduction of immunosuppression (MMF 1 g/d) was performed and an antitoxoplasma treatment (malocide + sulphadiazine) was started. One month later, due to the absence of any clinical improvement with the treatment, a stereotactic cerebral biopsy was carried out. 

**Figure 2:**
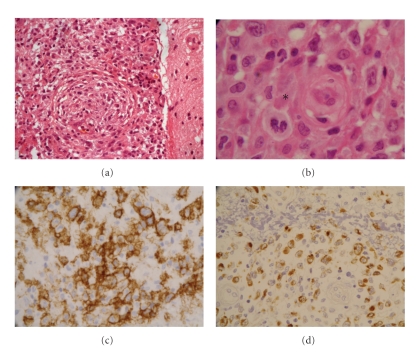
Cerebellar biopsy: (a) nodular granulomatous lesion with mononuclear cells (Hematein-eosin × 40). (b) Atypical cells with mitosis (*) (Hematein-eosin × 40). (c) Staining with anti-CD20 (×40): atypical cells are stained. (d) Staining with anti-EBV-LMP1 (×40): a lot of cells are stained.

**Figure 3:**
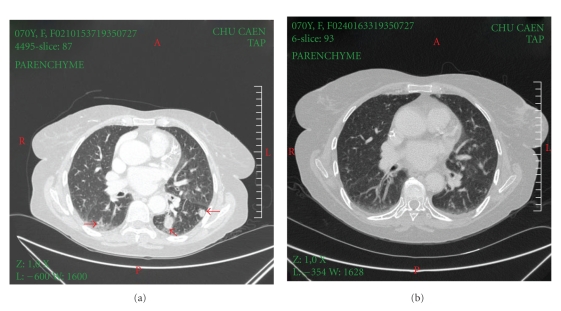
Pulmonary localisation of lymphomatoid granulomatosis. Transverse CT view, with contrast injection in parenchymatous window showing nodular lesions taking contrast. (a) At initial diagnosis. (b) 2 years after reduction in the immunosuppression and rutiximab therapy.

 The histological study (Figure 2) showed a heterogeneous necrotising lesion, with cellular remnants, granulomatous clusters of giant cells, with circumvented nuclei and well visible nucleoli, which looked like lymphoplasmocyte cells. The cells created dense castings on the meninges and were infiltrating the wall of several vessels. The immuno-histochemical study showed granulomatous cells stained by antibodies (ab) anti-CD68 (macrophages), anti-CD20 (B cells), and anti-CD30. The reaction to anti-EBV ab (anti-LMP1) was positive; conversely, the reaction to antitoxoplasma ab was negative. In view of these results, we concluded a grade III lymphomatoid granulomatosis according to LIPFORD classification [3, 4]. A complete workup including chest and abdominal CT was carried out. The CT scan of the thorax showed 6 pulmonary nodules of tissular density in the left lung and one pulmonary nodule in the right lung (Figure 3(a)). The CT of the abdomen was free. The bronchial endoscopy revealed purulent secretions with severe inflammatory reaction. Bronchoalveolar lavage was negative for Koch's bacillus. The histological study showed unsteadily scraped bronchial mucous membrane. The subjacent chorion was composed of an inflammatory polymorphic infiltrate, rich in polynuclear eosinophils with small growing granulomas. The diagnosis of grade III LYG with pulmonary and cerebral localization was considered. So, the immunosuppression was again minimized. MMF was discontinued; tacrolimus was reduced to 1 mg/d, to achieve trough levels around 3 ng/mL, whereas prednisone was increased to 0.5 mg/kg/d. 

Two months following immunosuppression reduction, the lesions were persistently unchanged. A treatment by anti-CD20 antibodies was then initiated (Rituximab: 375 mg/m^2^ weekly for 4 weeks). Markedly, after the first two doses, a significant clinical improvement was noted. The tolerance of rituximab was good. Six months later, the patient reported a complete disappearance of headache and a significant regression of gait disorders. Two years later (December 2007), there was no clinical sign of recurrence, the renal function was stable, and the brain MRI showed the persistence of hypersignals and infarction zones remnants. The thoracic CT scan showed only a single nodule (Figure 3(b)). The immunosuppressive treatment consisted of tacrolimus 1 mg/d and prednisone 0.2 mg/kg/d.

The last thoracic and brain CT scans (February 2010) showed a complete disappearance of the pulmonary and cerebral lesions. Laboratory results revealed creatinine level 1.1 mg/dL (101 *μ*mol/L), negative proteinuria, normal LDH level, and negative EBV plasmatic PCR. Immunophenotyping of blood lymphocytes revealed low CD19 count (10/mm^3^). 

## 3. Discussion

This paper highlights the diagnostic and therapeutic difficulties of LYG. Our patient presented many risk factors of this disease including: age, immunosuppression, administration of antilymphocyte sera for her two renal transplants, and the reactivation of CMV. The clinical presentation demonstrated the heterogenecity and the abundance of the symptoms. It should be noted a clinicoradiological delay for the diagnosis of pulmonary lesions. Our therapeutic approach was sequential, initially by a reduction in the immunosuppression, then rituximab was introduced.

### 3.1. Frequency

LYG is a rare disorder belonging to the group of type B lymphoproliferative diseases. It affects males > females (2 men for 1 woman) and affects primarily immunocompromised patients. The peaks of frequency concern 3rd and 6th decades [5, 6]. Patients presenting LYG have 10 to 60% risk to develop large B cells lymphoma. LYG is a serious disorder with a median survival of 2 years in the general population. The main cause of death is the progression of pulmonary lesions [7].

In renal transplantation, the literature reported 6 cases among which 3 cases were diagnosed postmortem and 3 other cases with good outcome (one case after a reduction in immunosuppression, 2 cases after chemotherapy) [1, 2, 8–11].

### 3.2. Clinical Features

LYG can affect all organs and may simulate a systemic disease. Clinical presentation varies according to the localization of granulomatous lesions [12]. Lung involvement is present in approximately 90% of cases [13]. Cutaneous lesions (25–50% of cases) were also reported, as well as central nervous system lesions (25–35% of cases) with multiple and focal localizations [14]. Other organs can be affected: the kidneys (20 30%), the liver (20 30%), and, less frequently, gastrointestinal tract [8, 12].

### 3.3. Diagnostic Difficulty

Due to its rarity, its various clinical presentations, and the absence of clinical, biological, or radiological specificity, the recognition of LYG is usually complex. This explains the delay of the diagnosis which can go up to 3 to 6 months after the beginning of the symptoms. Unfortunately, the diagnosis is realised by autopsy in many cases [8–10]. 

### 3.4. Complementary Examinations

Blood analyses, lumbar puncture examination, and imaging assessments are not specific. Radiological examinations can localize the lesions, but often with a clinicoradiological lag [1]. Brain MRI is the examination of choice for cerebral lesions. The lesions are often multiple, with T2 hyperintense signal. The most characteristic aspect is the punctiform or linear pattern of enhancement [14]. After treatment, an enhanced T2 can persist; however, a complete disappearance of the cortical infarction zones can be reached, as happened in our patient. The thoracic CT scan demonstrates multifocal infiltration in the lungs, predominating the lower lobes, with variable radiological aspects: reticulonodular alveolar opacities, pleurisy, or nodules [15, 16].

### 3.5. Histopathological Examination

Three-tiered grading system (I, II, and III) have been proposed by Lipford et al. for LYG on the basis of cellular atypia and degree of inflammatory background. This classification predicts the response to treatment and the survival [3, 17]. Nevertheless, it is important to note the difficulty of the histopathological diagnosis, as the presence of wide zones of inflammation and necrosis may suggest an infectious origin (such as toxoplasmosis) rather than LYG.

Our patient was grade III. The histological analysis showed an angiodestructive polymorphous lymphoid infiltrate. The cells were overwhelmed within important necrotising zones and altered inflammatory residues. They were gathered in granulomatous clusters with lymphoid abnormal cells of type B and lymphoplasmocyte cells. There was an infiltration of the vascular wall with intravascular thrombi. The immunophenotyping illustrated a positive stain by anti-CD20+ ab (B cells), anti-CD68+ ab (macrophages), anti-CD30 ab, and EBV-LMP1+ ab revealing EBV infection.

### 3.6. Risk Factors

EBV is the principal trigger factor of lymphoproliferation (especially in cases of donor EBV+/recipient EBV−). EBV viral load is considered to play a key role in mediating the disease process [4]. Infection with CMV, which occurred in our patient, may also contribute in lymphoproliferation development. The over-immunosuppression is supposed to be responsible, in particular the cumulative doses of antilymphocyte sera. Moreover, the impact of other factors as age and cigarette smoking have been also reported [4, 18].

### 3.7. Management

There are scanty data in the literature concerning the management of LYG. In our patient, we considered that it might be beneficial to reduce calcineurin inhibitors by 50% and withdraw antiproliferative medications (mycophenolate or azathioprine) based on data previously reported in overall PTLD [1, 19].

An early trial showed improved survival with therapy comprising corticosteroids and cyclophosphamide, but mortality of LYG remained very high [20]. Combined chemotherapy using CHOP protocol (cyclophosphamide, doxorubicine, vincristine, and prednisone) was also proposed [5]. 

More recently, few reports have tried a targeted therapy with the anti-CD20 antibody rituximab with or without traditional combined chemotherapy. To date, a total of 12 cases were published in the literature. The responses were successful in 9 cases [6, 13, 21–27], or ineffective in 3 cases [28–30]; however, none of those series concerned renal transplant patients.

## 4. Conclusion

LYG diagnosis is complex because of the diversity of its clinical presentation, the nonspecific imaging aspects, and the difficulty of histopathological reading. To be successfully treated, this malignant disorder requires an early management. Our report showed that rituximab could be considered a valuable option for the treatment of LYG in renal transplant patients.
